# Authors’ reply to: ‘Stent-free is not risk-free: risk-defined DAPT after DCB-only PCI in ACS’

**DOI:** 10.1093/ehjcvp/pvag059

**Published:** 2026-08-04

**Authors:** Anton Håkansson, Sacharias von Koch, Axel Dahlgren, Christian Reitan, Sasha Koul, Stefan James, Tomas Jernberg, Per Grimfjärd, Elmir Omerovic, Oskar Angerås, David Erlinge, Moman A Mohammad

**Affiliations:** Division of Cardiology, Department of Clinical Sciences Lund, Lund University, BMC I12, 221 84 Lund, Sweden; Division of Cardiology, Department of Clinical Sciences Lund, Lund University, BMC I12, 221 84 Lund, Sweden; Division of Cardiology, Department of Clinical Sciences Lund, Lund University, BMC I12, 221 84 Lund, Sweden; Department of Clinical Sciences, Cardiology, Karolinska Institutet, Danderyd Hospital, 182 88 Stockholm, Sweden; Division of Cardiology, Department of Clinical Sciences Lund, Lund University, BMC I12, 221 84 Lund, Sweden; Department of Medical Sciences, Uppsala University, 751 85 Uppsala, Sweden; Department of Clinical Sciences, Danderyd Hospital, Karolinska Institutet, 182 88 Stockholm, Sweden; Department of Medical Sciences, Västmanland Hospital, 721 89 Västerås, Sweden; Division of Cardiology, Department of Molecular and Clinical Medicine, Sahlgrenska Academy, University of Gothenburg, 413 45 Gothenburg, Sweden; Department of Thoracic Surgery and Cardiology, Sahlgrenska University Hospital, 413 45 Gothenburg, Sweden; Institute of Medicine, Sahlgrenska Academy, University of Gothenburg, 413 90 Gothenburg, Sweden; Division of Cardiology, Department of Clinical Sciences Lund, Lund University, BMC I12, 221 84 Lund, Sweden; Division of Cardiology, Department of Clinical Sciences Lund, Lund University, BMC I12, 221 84 Lund, Sweden

## Authors’ answer

We thank the authors for their careful reading of our study. We agree that all three suggestions would be informative. Each is limited by the same problem: a small sample, particularly in the abbreviated arm (*n* = 141), and a pre-specified Holm–Bonferroni plan under which all analyses beyond the primary composite are exploratory.

### Component-specific absolute risk differences

We agree that absolute risks are what inform clinical decisions, and we report a doubly robust absolute risk difference for the composite endpoint [augmented inverse probability weighting: +5.6 percentage points, 95% confidence interval (CI) −2.9 to 14.1]. We chose not to extend this to each component. The component event counts are low and uneven between arms (death 6 vs. 20, myocardial infarction 17 vs. 88, stroke 1 vs. 6, bleeding 8 vs. 41). Absolute risk differences from counts this small would have very wide CIs, and because they sit below the primary endpoint they cannot support confirmatory claims. The crude and weighted event percentages are already given in Table 2. We judged that a formal component-by-component presentation would carry a high risk of being over-interpreted.

### Within-stratum weighting

The commentary is correct that the de novo and in-stent estimates were obtained under the overall weights, without re-weighting within each lesion stratum (Figure 3 legend), and we agree this warrants clarification. Effect modification was assessed with a pre-specified treatment × lesion-type interaction term, which was not significant. This test is underpowered for the same reasons mentioned before, so it does not exclude heterogeneity. The stratum-specific hazard ratios are descriptive, and we have tried to clearly label the in-stent restenosis signal as exploratory. We did not re-estimate propensity weights within strata, as we believe that the exceedingly low event rates within the abbreviated arm would make the weights and their diagnostics unstable. However, to let readers judge balance under the applied weights, standardized mean differences within each lesion stratum are shown in Supplementary material online, *Figure S1*.

### Achieved antiplatelet exposure

We fully agree, and consider this the most important of the three points. We agree that the intention-to-treat variables in non-randomized registries do not have the same protection as randomization. However, a non-adjudicated per-protocol analysis in an observational setting such as this is not protected against misclassification of exposure bias either. Given the scarcity of data on this topic, we considered the available intention-to-treat data and the findings sufficiently valuable to report. As stated in the paper, we had no data on the treatment regimen actually received by the patients; our adherence figures (∼80% adherence, ∼5% crossover) were taken from other Swedish acute coronary syndrome cohorts. As this study exclusively examined ticagrelor, the overall rate of non-adherence, driven in part by ticagrelor-specific side effects such as dyspnoea, may be higher than in studies employing mixed antiplatelet regimens. However, there is no compelling reason to expect this to affect one treatment arm disproportionately. Therefore, we believe that non-adherence would likely result in dual antiplatelet therapy shortening symmetrically across both groups, limiting its impact on between-group comparisons.

We would again like to thank Xiang Zhou and colleagues for their insightful comments, and hope that they find our reasoning to be acceptable.

*Sincerely, on behalf of the authors, Anton Håkansson and Moman Mohammad*

## Supplementary Material

pvag059_Supplementary_Data

## Data Availability

The data used in this study are held by the Swedish National Board of Health and Welfare and SWEDEHEART and cannot be shared publicly due to national legal restrictions.

