# Supplementary material for: Authors’ reply to: ‘Stent-free is not risk-free: risk-defined DAPT after DCB-only PCI in ACS’
Source: Eur Heart J Cardiovasc Pharmacother. 2026 Aug 4;12(7):592–3. doi: 10.1093/ehjcvp/pvag059 (PMC13730585; doi:10.1093/ehjcvp/pvag059)
Supplement: Supplementary file 1 [file pvag059_supplementary_data.docx]

**Supplementary figure 1. Within lesion-stratum SMD distribution after IPT-weighting**

**Figure S1.** Absolute standardized mean differences (SMDs) for each covariate in the propensity-score model, shown before (Unweighted) and after (IPTW) weighting, within the two culprit-lesion strata: (A) de novo lesions (abbreviated n = 84, standard n = 555) and (B) in-stent restenosis (ISR) lesions (abbreviated n = 57, standard n = 432). Weights are the inverse-probability-of-treatment weights estimated once in the full cohort and applied without re-estimation within strata; each panel restricts both the sample and these weights to the corresponding stratum. Points show the absolute SMD per covariate; circles denote the unweighted sample and triangles the IPTW-weighted sample.
NSTEMI, non-ST-segment-elevation myocardial infarction; STEMI, ST-segment elevation myocardial infarction; DAPT, dual antiplatelet therapy; IPTW, inverse-probability-of-treatment weighting; SMD, standardized mean difference.
